# Automated sub-5 nm image registration in integrated correlative fluorescence and electron microscopy using cathodoluminescence pointers

**DOI:** 10.1038/srep43621

**Published:** 2017-03-02

**Authors:** Martijn T. Haring, Nalan Liv, A. Christiaan Zonnevylle, Angela C. Narvaez, Lenard M. Voortman, Pieter Kruit, Jacob P. Hoogenboom

**Affiliations:** 1 Department of Imaging Physics, Delft University of Technology, Delft, The Netherlands; 2 Delmic BV, Delft, The Netherlands

## Abstract

In the biological sciences, data from fluorescence and electron microscopy is correlated to allow fluorescence biomolecule identification within the cellular ultrastructure and/or ultrastructural analysis following live-cell imaging. High-accuracy (sub-100 nm) image overlay requires the addition of fiducial markers, which makes overlay accuracy dependent on the number of fiducials present in the region of interest. Here, we report an automated method for light-electron image overlay at high accuracy, i.e. below 5 nm. Our method relies on direct visualization of the electron beam position in the fluorescence detection channel using cathodoluminescence pointers. We show that image overlay using cathodoluminescence pointers corrects for image distortions, is independent of user interpretation, and does not require fiducials, allowing image correlation with molecular precision anywhere on a sample.

Advances in fluorescence microscopy (FM) have enabled protein localization with an accuracy of tens of nanometers^1^. However, FM does not reveal the subcellular ultrastructure, which is done by electron microscopy (EM). Data obtained with correlative light and electron microscopy (CLEM) provides unique insight in biological structure-function relations and allows both ultrastructural determination after live-cell imaging and fluorescent determination of biomolecules^2^,^3^. Correlation is challenging because the contrast mechanisms and the magnification in both modalities are widely different. Integrated microscopes^4^,^5^,^6^ facilitate CLEM and several systems have been recently reported^7^,^8^,^9^,^10^,^11^,^12^ and became commercially available. Even for integrated microscopy, retrieval in EM of a region of interest (ROI) identified previously in FM requires the presence of fiducial markers that are discernible in both microscopes. These markers may appear endogenous, such as cell patterns^13^, but these are only occasionally present and their size precludes high-accuracy image registration. Exogenous markers such as gold nanoparticles or quantum dots^14^ can offer FM-EM image overlay at 50–100 nm accuracy^15^,^16^,^17^,^18^, but only when the number of fiducials present is sufficient in the selected ROI. Moreover, identification of a region of interest requires additional pattern recognition procedures. FM-EM registration procedures that do not rely on fiducial-markers and that can generate an overlay with a priori known accuracy, irrespective of the location of the ROI on the sample, could greatly simplify CLEM, allowing routine inspection, precise, molecular correlation, and enhanced throughput.

Below, we present an automated procedure for FM-EM registration and generation of a correlative overlay image in tissue and whole cell CLEM samples. Our method relies on the concept of cathodoluminescence (CL) pointers: the electron-beam generates light that is recorded in the FM channel of an integrated microscope with nanometer accuracy. This CL pointer procedure can be automatically applied after recording FM and EM images of the ROI, is independent of user interpretation and fiducial markers, and accounts for distortions between FM and EM image coordinate frames.

## Results

**Figure 1:**
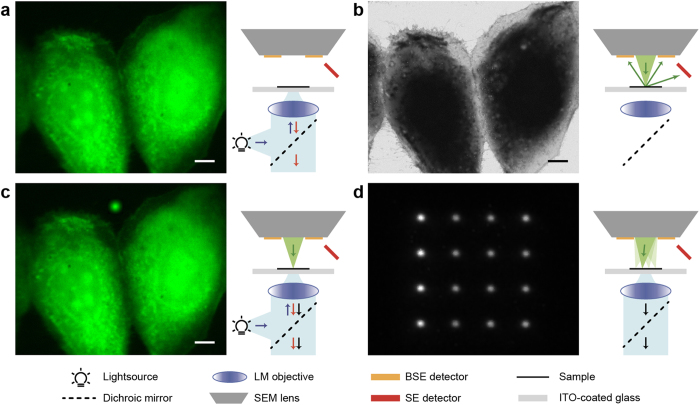
Working principle of cathodoluminescence for automated alignment. (**a**) Fluorescence microscopy of MDCK cells expressing paxilin-GFP performed *in-situ* in a SEM. (**b**) The same area visualized with SEM. (**c**) Fluorescence microscopy as in (**a**) but with simultaneously the electron beam in spot mode. Due to substrate cathodoluminescence (CL), the position of the electron beam can be detected in the optical detection channel. (**d**) By quickly alternating the position of the electron beam to different locations in spot mode, multiple CL pointers can be detected simultaneously in a single acquisition using the light microscope. SE, secondary electrons; BSE, back-scattered electrons; ITO, indium tin oxide. Scale bars are 10 μm.

Integrated microscopes can allow for the simultaneous observation of a region on a sample with both FM and EM^11^,^12^,^19^,^20^. For our experiments, we have used a system, in which epi-FM is integrated in a Scanning EM (SEM)^10^,^19^ (see also schematics in Fig. 1). The FM illuminates and observes the sample through a transparent substrate, while SEM imaging occurs from above the sample. With this configuration, CLEM can be performed on both glass-mounted tissue sections, as well as whole cells cultured and fixated directly onto the substrate^19^,^21^. We illustrate the detection of electron-beam generated CL in the fluorescence detection channel with a sample of GFP-labeled MDCK cells cultured on a conductive ITO-coated glass slide. The FM image (Fig. 1a) shows the signal of GFP-tagged Paxilin expressed in two nearby cells. The same region is imaged in the SEM (Fig. 1b). Next, we put the electron beam at a stationary position and record an FM image simultaneously. An additional bright spot is observed (Fig. 1c) resulting from CL generated in the glass substrate. This CL spot thus indicates where the chosen electron beam (*x, y*)-position, and thereby a particular pixel from the SEM image, is projected in the FM image By deflecting the electron beam to another position, this temporary CL spot will appear elsewhere within the FM field of view. We will refer to these spots as CL pointers, which highlight a set position in the EM coordinate frame on the FM camera. Within the integration time of the FM camera, the electron beam can be positioned at multiple locations, leading to the simultaneous detection of an array of pointers (Fig. 1d) that can span the entire FM field of view.

**Figure 2:**
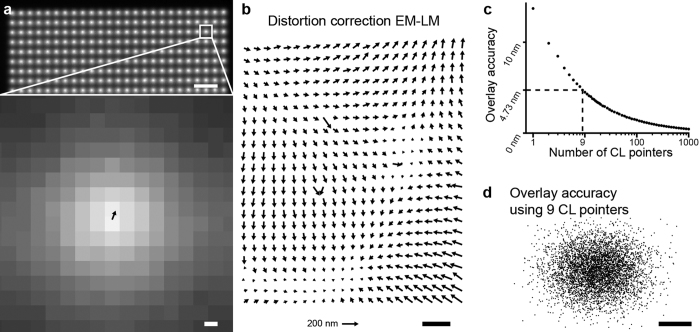
CL pointer array with retrieved distortion field and overlay accuracy. **(a)** Upper part of a 25 × 25 CL pointer array as recorded using the optical microscope. The magnified view shows the displacement vector δ between FM and transformed EM coordinates drawn to scale. **(b)** By averaging displacement vectors as shown in (**a**) over multiple acquisitions, a distortion field between FM and EM coordinates is calculated. **(c)** The estimated overlay accuracy versus the number of CL pointers shows a predicted N^−1/2^ behavior. For this experiment, the accuracy is <5 nm for ≥9 CL pointers. **(d)** Distribution of overlay errors using different sets of 9 CL pointers. Scale bars are (**a**-top and **b**) 10 μm, (**a**-bottom) 200 nm and (**d**) 5 nm. The scale for the length of the distortion vectors in (**b**) is indicated by the arrow at the bottom indicating 200 nm distortion.

The intensity distribution of a CL pointer is circularly symmetric (Fig. 2a) with its size determined by the electron interaction volume convolved with the point spread function of the FM. The full width half maximum (FWHM) of the CL pointer typically spans from around 300 nm (the optical diffraction limit, for beam energies <5 keV or lower) to several micrometers. Figure 2a shows part of a recorded dense pointer array with spacing of 4 μm, i.e. about 1.4 times the FWHM, leading to 625 pointers in total. The circular symmetry allows for rapid and accurate center localization for each pointer^22^,^23^,^24^. In this way, for each pointer, a retrieved location in the FM image coordinate frame can be paired with the corresponding coordinate in the SEM image frame. We next determine the linear transformation *T* (i.e., including translation, rotation, and scaling) between both sets of coordinates. For each pointer *i*, we then define the difference vector *δ*_*i*_ between original and transformed coordinates:


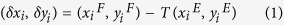


where superscript *F* denotes coordinates in the FM frame, and *E* in the EM frame. Note that the choice of either FM or EM as the frame of reference is arbitrary for our purpose of registering both images for data correlation. The arrow in the magnified CL intensity profile in Fig. 2a denotes such a difference vector.

For each vector 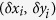, magnitude and orientation depend on the error introduced in FM center localization plus the non-linear image distortions in both microscope systems. Averaging over a set of sequentially obtained pointer arrays allows us to determine the distortion profile (Fig. 2b) over the full FM field of view. These distortions arise in part from the EM as we see changes in the distortion pattern when varying the EM settings, most notably electron energy (data not shown). Determining the exact origin of these distortions requires further investigation. We note that the magnitude of the observed distortions are all within the specified performance of our EM. At three positions in the distortion profile, vectors deviant in magnitude and orientation from the overall gradual pattern are observed. As these are observed consistently for all EM settings as well as for different FM objectives, we attribute these to the FM camera pixel array.

The distortion profile determined in Fig. 2b remains constant as long as the EM and FM microscope alignment are left unchanged. Thus, for a series of measurements, this profile has to be determined only once after which it can be used to correct each collected image pair, including after sample translation for crossing to different areas. In a typical measurement, a high resolution EM image is recorded from a selected area within the FM field of view. For each such measurement, only the exact position and orientation of the EM image in the FM field of view and their relative scaling then still has to be determined to obtain the image registration. This requires exposure of a single CL pointer array within the field of view of the just-captured EM image, a procedure that can typically be completed in a few seconds. We note that all the steps from exposing and recording the CL pointer array to generating the overlay image can be performed in a fully automated fashion after recording the SEM image, requiring no additional user interaction or interpretation. Only a change in EM, or possibly FM, magnification would need a re-evaluation of the distortion pattern, which can be performed as part of the re-focusing procedure. For high-throughput applications, we would thus recommend a priori determination of optimized EM magnification and imaging settings after which automated overlay can be generated for each image pair.

We use similar CL pointer arrays to evaluate the overlay accuracy of the above detailed procedure. First, we determine the FM-EM distortion profile. Next, we consecutively expose 50 arrays that consist of 625 pointers each for a total of 31250 pointers, and correct their position using the determined distortion profile. To evaluate the overlay accuracy as a function of the number of pointers *N* used in the procedure, We randomly select a subset of *N* pointers from the total set. We use these *N* pointers to estimate the FM-EM registration. Using this registration, we transform the center position of the EM image coordinate system to FM coordinates. By repeating this 5000 times for different (random) selections of *N* pointers, a distribution of center positions is obtained. The standard deviation of this distribution is an estimate of the overlay accuracy when using *N* pointers. Figure 2c shows this overlay accuracy as a function of the number of CL pointers, which follows an expected *N*^−1/2^ behavior. We see that we achieve a sub-5 nm overlay accuracy for 9 CL pointers which decreases further to negligible values with increasing array size. Figure 2d shows the resulting spread in overlay errors for *N* = 9.

**Figure 3:**
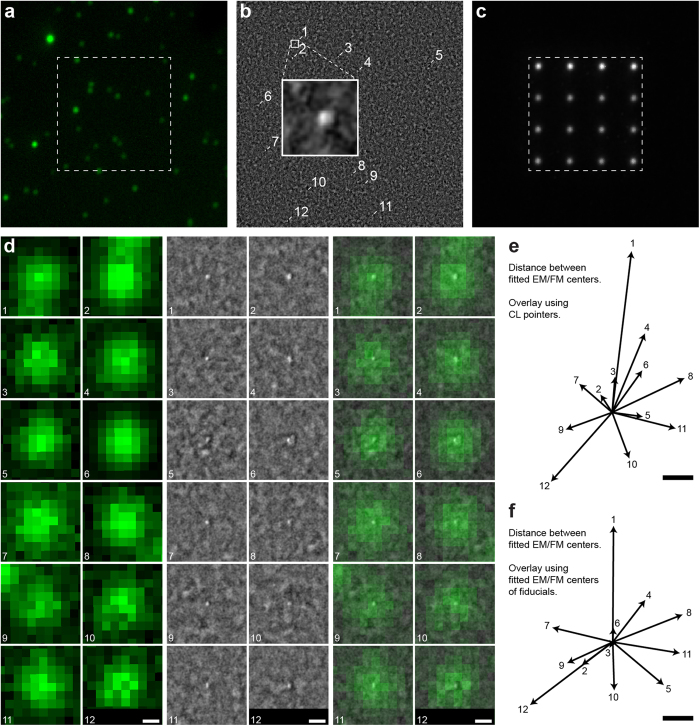
Validation of CL pointer overlay using correlative fiducials. **(a)** Fluorescence image of dye-doped SiO_2_ nanospheres. **(b)** Scanning electron microscopy image of marked area in (**a**), inset shows a magnified view of an isolated nanosphere. **(c)** CL pointer array used to align FM (**a**) and EM (**b**) images, imaged area is equal to (**a**) and marked area equal to area in (**b**). **(d)** Magnified view of FM (left), EM (middle), and overlaid FM + EM (right) images from (**a**) and (**b**) for each particle numbered in (**b**). Overlay determined using the CL pointer array shown in (**c**). **(e)** Difference vectors between fitted EM to FM centers for each of the spheres in (**f**). **(f)** Difference vectors as in (**e**) but with overlay calculated using the nanospheres in (**a**) as fiducial markers instead of using CL pointers. Scale bars are (**d**) 200 nm and (**e,f**) 10 nm.

To illustrate the overlay procedure and the above achieved registration accuracy, we provide an example using a sample comprised of typical fiducial markers, fluorescence labelled silica nanospheres. The 40 nm diameter nanospheres were spin-coated at low concentration on an ITO-coated cover glass. Figure 3a shows a FM image of the nanospheres recorded. Individual spots with varying fluorescence intensity can be observed, where the brighter spots presumably originate from small clusters containing multiple nanospheres. We select an area, indicated by the dashed square in Fig. 3a, containing mostly single spheres as judged from the observed intensities. Figure 3b shows the SEM image recorded from this area where we have indicated each sphere with number index for clarity. The CL pointer area recorded consecutively within this same SEM field of view is shown in Fig. 3c. Completing the registration procedure detailed above, we obtain an overlay image. Figure 3d shows for each nanosphere the FM, SEM and overlaid images, visually illustrating the precise correlation over the entire field of view between SEM and FM nanosphere positions obtained with our registration procedure.

In order to assess the achieved registration accuracy, we fit the center positions of each nanosphere in both FM and SEM coordinate frames. We now evaluate the difference vectors between these positions after image registration, similar to our procedure for the CL pointers. Figure 3e shows the set of obtained difference vectors, with numbering corresponding to the indices defined in Fig. 3b. Note that signal to background for the nanospheres in the SEM is relatively low compared to the FM. Also, several spheres do not appear to be spherical (e.g., particles 2, 4, 11, and 12), have bad signal to noise ratio (e.g., particle 5), or, based on the brightness in FM, to actually consists of multiple nanospheres (e.g., particles 6). This most likely provides a dominating contribution to the observed difference vectors.

Now, in order to compare the accuracy of our automated registration procedure to one based on fiducial markers, we compose an alternative overlay image using the nanospheres as fiducial markers. This second image registration is based on the sets of fitted nanosphere center positions in FM (Fig. 3a) and SEM (Fig. 3b) coordinate frames. Following the same procedure as for the CL pointer registered images, we then construct the difference vectors remaining for the fiducial-marker-based overlay. The resulting vectors are plotted in Fig. 3f. As can be seen, the differences between the results obtained for automated overlay (Fig. 3e) versus fiducial overlay (Fig. 3f) are all within about 10 nm. Note that in Fig. 3f the fiducials are used both for the registration and the estimation of the overlay errors. Thus, the vectors plotted in Fig. 3e and f all provide an estimate of the intrinsic localization errors. Using higher numbers of CL pointers and/or more fiducials both vector plots would become even more similar. Nevertheless, we conclude that our procedure provides results comparable to fiducial-based overlay with >10 fiducials, with the additional advantages that our methodology can be applied anywhere on the sample, throughout the entire FM field of view, with an overall constant, pre-determined accuracy given in Fig. 2c. Moreover, this registration accuracy is such that fluorescence localization within the EM structural image, is mainly limited by the intrinsic errors in localization within the SEM and FM images and not by how the image overlay is performed. Note that the fiducials used in this experiment only serve as a way to estimate the registration accuracy of our CL pointer based registration. Even though different experimental conditions and/or different fiducial markers might influence the accuracy of the fiducial marker registration, this only influences the lower-bound with which we can assess the CL pointer registration accuracy.

**Figure 4:**
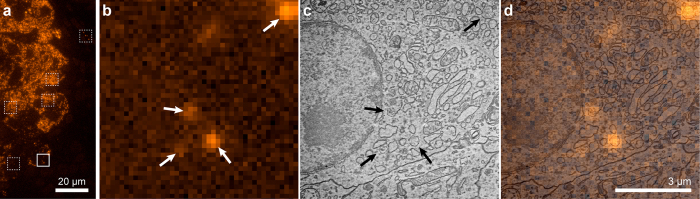
Example application of CL pointer alignment. Thin section of rat pancreas with post-embedding immunolabeling for insulin (Alexa594, orange). **(a)** FM, boxed areas indicate regions of interest selected for SEM, based solely on FM appearance in terms of either isolated or dense fluorescence. Data from dashed boxed areas is shown in Supplementary Fig 1. **(b)** Magnified view of solid boxed area in (**a**), followed by **(c)** scanning EM and **(d)** overlay. Arrows indicate isolated fluorescence features and the corresponding areas in SEM determined after generating the overlay. In the overlay, fluorescence appears well centered on distinct ~100–200 nm granules, while these granules do not a priori stand out in the SEM image. See Supplementary Fig. 1 for further data. Scale bar is 20 μm in (**a**), 3 μm in (**b–d**).

Where the above example illustrated the high registration accuracy that the CL pointer procedure can reach, a major advantage of this technique is that it can be applied anywhere on a sample with similar overlay precision. To provide an application example on biomedical tissue, we imaged a sample of pancreatic tissue section from a diabetes-prone rat^25^. The 70 nm tissue section is immuno-labelled after embedding in epon, with Alexa594 for insulin^26^. Sections are mounted on ITO-coated glass^27^ for inspection in the integrated microscope. Figure 4a shows a FM image of a selected part of the section where an endocrine part with insulin-producing beta cells (orange indicating Alexa594) is visible. We select several ROI for EM (boxed areas), which serve to illustrate biological validation of the overlay as well as applicability on arbitrarily selected (i.e. not depending on presence of registration markers) areas. The ROI shared between SEM and FM for the solid boxed area is shown in Fig. 4b (FM) and Fig. 4c (SEM). This area shows only isolated fluorescence indicated with arrows. The CL pointer registration procedure is automatically conducted after recording the SEM image, providing us with a precise mapping of the EM to FM coordinates. This results in the overlay image given in Fig. 4d (FM + SEM). We can clearly see that the Alexa594-insulin signal centers on 100–200 nm diameter granules that are discernible in the SEM image (indicated with arrows). Thus, the overlay image confirms biological interpretation of FM and SEM images of insulin-containing granules, with overlay accuracy well below 50 nm. However, no prior interpretation of SEM image, or user action was required and the overlay image was constructed fully automated immediately after recording the SEM image. As a result, any ROI on the sample can be overlaid, with, for all overlays, the same precision. In Supplementary Figure 1, we show the FM, SEM, and overlay images obtained for all dashed boxed areas in Fig. 4a. These include more data with isolated features, showing the identification of and centering on Alexa594-labeled granules, as well as crowded areas with many granules.

## Discussion

Various types of integrated microscopes have been reported for SEM and TEM both as early as the 1980’s 6 as well as in recent years^4^,^5^. Our approach of detecting electron-beam generated CL pointers with the integrated FM can be incorporated in those microscopes where a common field of view is shared between the two modalities^8^,^9^,^10^,^11^,^12^. The principle of CL pointers was demonstrated above using ITO-coated glass, but any CL-generating substrate could be used. While glass or other optically transparent substrates are clearly needed in our integrated microscope^10^, silicon nitride membrane grids with a few tens of nanometers of CL active material^28^ could be used in integrated (scanning) transmission electron microscopes^12^. Apart from whole cell investigation^19^,^21^, glass slides have been used as support samples for thin tissue sections in CLEM, particularly in the combination with optical superresolution^29^ or 3D analysis using serial sections in array tomography^30^,^31^. For both these applications, the registration procedure reported here may have strong benefits.

In superresolution-CLEM, a main aim is to achieve biomolecular localization within the EM recorded cellular ultrastructure, preferably with a precision approaching intrinsic EM resolution or bio-molecular length scales. Sample distortions arising during intermediate preparation steps, e.g. sectioning or staining for EM, affect FM-EM overlay accuracy with inspection on stand-alone microscopes, which can be mitigated with integrated inspection. The CL-pointer registration procedure provides certainty on the achievable overlay accuracy provided by the number of pointers used, which can be adjusted by the user (see Fig. 2c). Using fiducial markers this would be dependent on the (random) number of fiducials present around the ROI. Moreover, with CL-pointers the overlay error in CLEM can be reduced to <5 nm, which can be an order of magnitude lower than typical localization accuracy in FM (10–50 nm). Hence, the resolution of FM will then be the dominant contribution to how accurate a molecule or cluster of molecules can be localized in an EM image. In addition, approaching nanometer-scales in overlay, machine-specific issues such as stage drift may also affect the registration. With a strongly CL emissive substrate like glass, recording the CL pointer arrays can be conducted in a few seconds, as for the examples given above. In our microscope, this translates to well below 1 nm drift during CL pointer acquisition. This means the overall measurement time is limited by FM and SEM image integration times. Then, drift correction needs to be conducted on both the FM and SEM imaging systems separately, which can be done using existing drift correction procedures. We note that as our CL pointer procedure registers FM and SEM coordinate frames, irrespective of sample drift, it could also be used to monitor SEM to FM drift in the integrated microscope over longer observation times. We also note that besides long image integration times, where drift correction needs to be conducted, also very fast scanning may cause artefacts: the precision with which spot-mode CL pointer positions in the SEM match the corresponding positions in scanning mode would then need to be considered. For most standard SEM’s and typical imaging conditions for biological specimen, like we used here, this is however not an issue.

Our registration procedure relies on the use of an integrated microscope. With integrated microscopy, potential sample deformation between FM and EM measurements is avoided which is essential for achieving a high accuracy overlay. Several types of integrated CLEM microscopes have appeared in recent years^4^,^7^,^8^,^9^,^10^,^11^,^12^, most of which allow CL detection needed to record registration pointers. However, it also requires ‘integrated’ sample preparation^2^, i.e., both the preparation for FM and for EM has to be conducted before inspection. Our work parallels the development of fixation-resistant probes^32^, sample preparation protocols maintaining fluorescence in resin-embedded sections^33^,^34^, efficient post-embedding on-section immuno-labelling^2^,^26^ (see Fig. 4), and strategies to preserve fluorescence in reduced vacuum conditions^35^. Together, these may indeed allow superresolution FM on EM-prepared tissue in an integrated microscope and construction of an integrated superresolution microscope is currently underway. With our CL pointer registration this may assist in enabling functional imaging with biomolecular precision within the ultrastructural context using CLEM.

The CL pointer registration can be performed in a fully automated fashion and in a few seconds. As Fig. 4 and Sup. Fig. 1 show, this can be carried out on arbitrarily selected regions with fluorescence expression. For automation, the crucial steps are the generation of CL pointer grids and the registration of EM to FM coordinates. As described below, we have achieved this using a combination of LabVIEW (National Instruments, US) and Matlab (The MathWorks, US), as well as using the open-source acquisition software Odemis (Delmic, the Netherlands). Automation is important in applications that involve large datasets acquired at a large number of locations^36^, such as for 3D 37 or large-scale 2D EM 25. A particularly promising application is 3D CLEM using automated array tomography, in which serial sections are imaged automatically to obtain a 3D volume reconstruction from the 2D images. Here, the ultimate prospect would be the development of fully automated, integrated correlative array tomography^3^, for which our automated, user- and fiducials-independent registration procedure may provide a crucial step forward.

In conclusion, we have presented a fiducial-marker free registration method for correlative light and electron microscopy. Our method uses cathodoluminescence pointers, i.e., detection of luminescence excited by the focused electron beam. We have demonstrated registering FM and EM coordinate frames with an accuracy below 5 nm, paving the way towards routine and unbiased high-resolution localization of bio-molecules using superresolution-CLEM. As CL pointer registration can be conducted fully automated anywhere on a sample, it may provide a crucial step towards high-throughput, high-resolution three-dimensional correlative reconstruction of biological samples using automated array tomography.

## Methods

### Sample preparation

All measurements were performed on ITO-coated microscope slides (thickness #1, 22 × 22 mm with 8–12 Ωsq^−1^ or 22 × 40 mm with 70–100 Ωsq^−1^; SPI Supplies, USA). The MDCK (Madin-Darby Canine Kidney Cells) epithelial cells shown in Fig. 1 were kindly provided by the Houtsmuller group at the Erasmus Medical Center (Rotterdam, the Netherlands), preparation is described in detail elsewhere^27^. The CL pointer array measurements shown in Fig. 2 use bare ITO-coated slides. Overlay validation measurements (Fig. 3) were obtained using 40 nm silica spheres loaded with a fluorescent ruthenium complex (SiFluor Ru 40 nm, carboxylated surface) (Active Motiv, USA), diluted and spin-coated on the microscope slides from a solution in water. Labeled grids (Fig. 4) were kindly provided by Pascal de Boer and Ben Giepmans (University Medical Center Groningen, Groningen, the Netherlands, with preparation described in Kuipers *et al*.^26^, using Alexa594 instead of quantum dots to label insulin. The embedded rat pancreas were redundant EM-samples from a previous study^25^. All methods involving animals^25^ were carried out in accordance with relevant guidelines and regulations: ‘The animals received humane care in compliance with the principles of laboratory animal care (NIH publication no. 85-23; revised 1985) and the Dutch law on experimental animal care. The university ethical board for animal studies approved all animal experiments reported in this study. The animals used in this study were fed ad libitum with food pellets conducted according to the guidelines for Ethics Committee and Animal Experimentation, University of Groningen’^25^.

### FM-EM integrated microscopy systems

Images where taken on two different integrated microscope systems. For Figs 1 to 3, data was collected on a home-built integrated microscope in an FEI Quanta FEG 200 SEM. The integrated wide-field fluorescence microscope, described in detail by Zonnevylle *et al*.^10^, was equipped with a Nikon 60 ×, 0.95NA CFI PLAN APO objective and 200 mm Nikon tube lens, an Andor Clara E camera for Figs 1 and 2 and an Andor Zyla 5.5 camera for Fig. 3. Data for Fig. 4 was collected using a SECOM platform (Delmic, the Netherlands) equipped with a Nikon 40x, 0.95NA CFI PLAN APO objective, installed on an FEI Verios SEM.

### Measurement details

The CL grid measurements (Fig. 2) were performed at 20 keV with a dwell time of 10 ms per CL marker. 50 arrays of 625 CL pointers each were captured sequentially. No dichroic or emission filters were used in this experiment. Measurements on the ruthenium-silica spheres were performed at 5 kV using 16 CL pointers with a 1 s dwell time per CL pointer and 134 seconds exposure for the EM image. The generation of CL pointer grids was automated using LabVIEW in combination with a data acquisition (DAQ) board (National Instruments, US). Fluorescence excitation was done using a Lumencor Spectra 4 at 485 nm center wavelength. The setup had a FF410/504/582/669-Di01 dichroic filter and a FF01-440/521/607/700 bandpass filter (both of Semrock, USA). No excitation filters were used besides those provided in the Lumencor light source. Fluorescence imaging of tissue sections (Fig. 4) was performed using the SECOM standard configuration (Delmic, the Netherlands), using the excitation with 555 nm center wavelength. SEM imaging was performed at 1 kV, in immersion-mode, with 30 μs dwelltime, at a working distance of 4.5 mm and using the Through-the-Lens Detector (TLD). Registration of EM to FM coordinates was performed fully automatically using the acquisition software Odemis (Delmic, the Netherlands) which uses 16 CL pointers in a 4 × 4 array.

### Analysis details

The position of the CL pointers and nanospheres in both FM and EM images was determined using the radial symmetry method of Parthasarathy^22^. To determine the linear transformation between the EM and FM CL pointer coordinates, a least squares solution is found for scaling, rotation and translation. Given


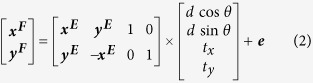


where 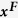, 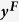 and 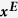, 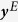 are column vectors of the optical coordinates and the electron coordinates respectively, 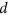 is the scaling, 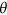 is the rotation, 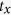 and 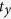 are the translation in x and y, and 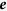 the residual vector, we minimize 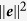 to find the rotation, scaling and translation between the EM and FM coordinate system. This transformation is carried out in Matlab (The MathWorks, US) using the cp2tform function. All the remaining distortions are characterized using the distortion profiles. Note that at least 2 pointer pairs are needed to calculate the transformation values. Since multiple CL pointers are detected in one optical exposure, the coordinates need to be sorted in order to create pairs of CL FM coordinates and EM coordinates. Using the outer edges of the grid, an initial guess is made for the grid position and pairs are found using a nearest neighbor algorithm. An iterative process of discarding and finding nearest neighbors is used to find each pointer pair, this method is insensitive to initial translation, rotation and scaling errors.

## Additional Information

**How to cite this article**: Haring, M. T. *et al*. Automated sub-5 nm image registration in integrated correlative fluorescence and electron microscopy using cathodoluminescence pointers. *Sci. Rep.* **7**, 43621; doi: 10.1038/srep43621 (2017).

**Publisher's note:** Springer Nature remains neutral with regard to jurisdictional claims in published maps and institutional affiliations.

## Supplementary Material

Supplemental Information
