# Supplementary material for: Automated sub-5 nm image registration in integrated correlative fluorescence and electron microscopy using cathodoluminescence pointers
Source: Sci Rep. 2017 Mar 2;7:43621. doi: 10.1038/srep43621 (PMC5333625; doi:10.1038/srep43621)
Supplement: Supplemental Information [file srep43621-s1.pdf]

Automated sub-5nm image registration in integrated correlative fluorescence and electron microscopy using cathodoluminescence pointers.

- Supplemental Information

Martijn T. Haring[1], Nalan Liv[1], A. Christiaan Zonnevylle[1], Angela C. Narvaez[1], Lenard M. Voortman[2], Pieter Kruit[1], and Jacob P. Hoogenboom[1*]

[1]Department of Imaging Physics, Delft University of Technology, Delft, The Netherlands.

[2]Delmic BV, Delft, The Netherlands

Supplemental Figure 1 | Further example application of CL pointer application to Figure 4. (a) Fluorescence image of thin section of rat pancreas with post-embedding immunolabeling for insulin (Alexa594, orange) as in Figure 4(a). Dashed boxed area indicates the region of interest selected for SEM with shown in Figure 4, solid boxed areas indicate regions of interest with data shown in panels **(b)**: magnified view of fluorescence, **(c)**: scanning electron microscopy, and **(d)**: automated overlay of fluorescence and scanning electron microscopy data.

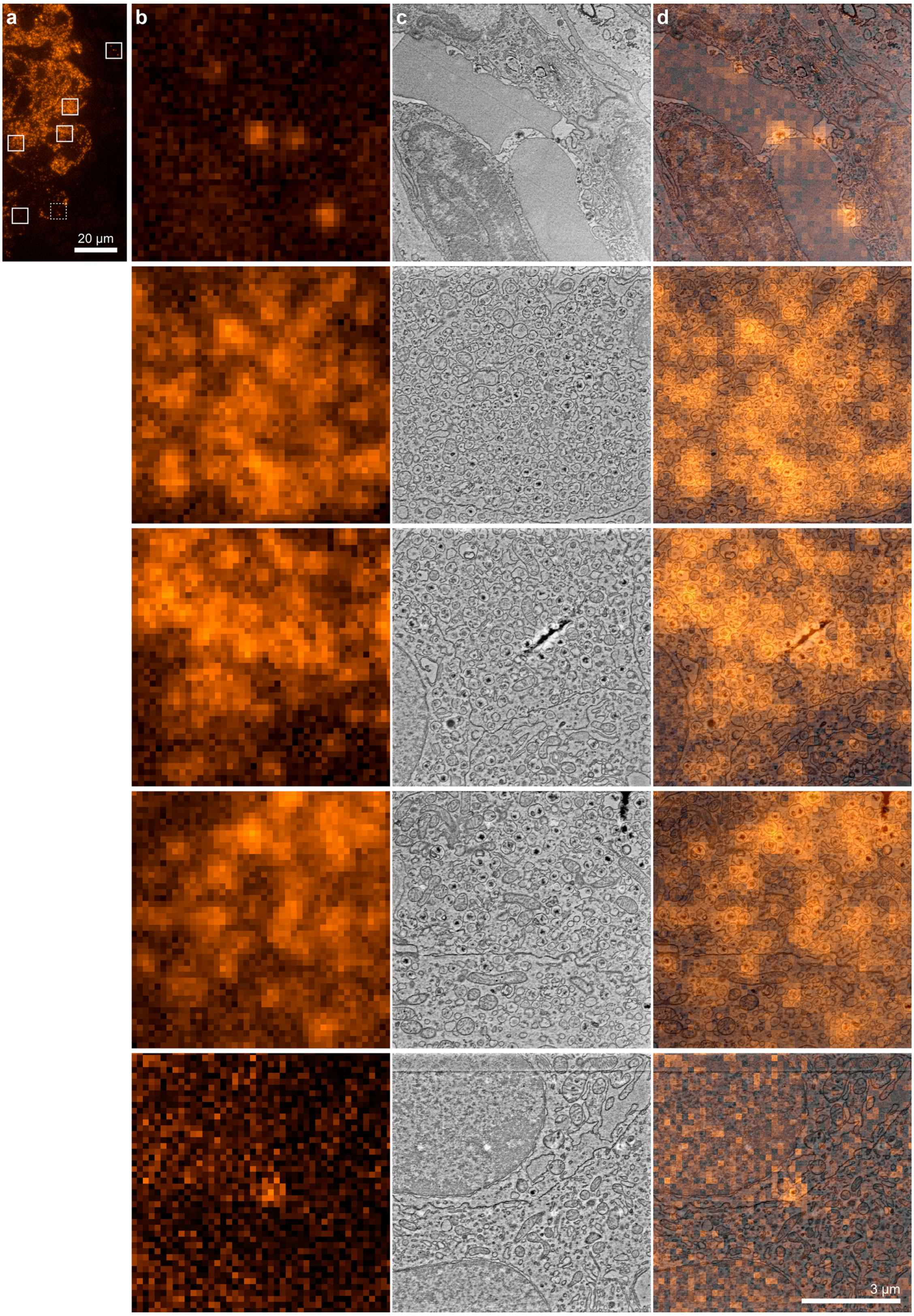
a
b
c
d
20 µm
3 µm
